# Inquiry diagnosis of coronary heart disease in Chinese medicine based on symptom-syndrome interactions

**DOI:** 10.1186/1749-8546-7-9

**Published:** 2012-04-05

**Authors:** Guo-Zheng Li, Sheng Sun, Mingyu You, Ya-Lei Wang, Guo-Ping Liu

**Affiliations:** 1 The Key Laboratory of Embedded System and Service Computing, Ministry of Education, Department of Control Science & Engineering, Tongji University, Shanghai 201804, China; 2 Laboratory of Information Access and Synthesis of TCM Four Diagnosis, Shanghai University of Traditional Chinese Medicine, Shanghai 201203, China

## Abstract

**Background:**

There is a long history of coronary heart disease (CHD) diagnosis and treatment in Chinese medicine (CM), but a formalized description of CM knowledge is still unavailable. This study aims to analyze a set of CM clinical data, which is important and urgent.

**Methods:**

Relative associated density (RAD) was used to analyze the one-way links between the symptoms or syndromes or both. RAD results were further used in symptom selection.

**Results:**

Analysis of a dataset of clinical CHD diagnosis revealed some significant relationships, not only between syndromes but also between symptoms and syndromes. Using RAD to select symptoms based on different classifiers improved the accuracy of syndrome prediction. Compared with other traditional symptom selection methods, RAD provided a higher interpretability of the CM data.

**Conclusion:**

The RAD method is effective for CM clinical data analysis, particular for analysis of relationships between symptoms in diagnosis and generation of compact and comprehensible symptom feature subsets.

## Background

Western medicine classifies coronary heart disease (CHD) as a kind of myocardial dysfunction and organic lesion, occasionally accompanied by coronary artery stenosis and vertebrobasilar insufficiency [1]. In contrast, Chinese medicine (CM) classifies CHD as a type of chest paralysis and heart pain, for which effective diagnosis and treatment are available [2].

CM treatment is based primarily on syndrome differentiation and physiology and pathology of *Zang-fu *organs and meridians. In CM, a symptom represents an observable indicator of abnormality, while a syndrome is the disease state manifested by symptoms. The connections between symptoms and syndromes in CM are not clearly defined. Therefore, it is necessary to delineate different relationships between symptoms and syndromes and explain the diagnosis results in comprehensible terms [3].

Machine learning builds empirical models on data for analysis and forecasting, which has recently been used for CM data analysis. Huang and Gao [4] reviewed several classifiers of data mining in CM. Li and Huang [5] used fuzzy neural network for analysis of CM ingredients. Wang *et al*. [6] used a decision tree method to generate prediction models for CM hepatitis data and liver cirrhosis data. Zhang *et al*. [7] combined factor and cluster analysis in the classification of CM syndromes related to post-hepatitic cirrhosis. Zhang *et al*. [8] used latent tree models to aid CM diagnosis. Knowledge discovery in database (KDD) [9], rough set [10], and expert system [11], have also been applied to CM.

Most CM machine learning works does not consider the medical meaning and links among features. However, CM data contain a large quantity of symptoms or syndromes which have specific medical meaning. Therefore, seeking the links between features including symptoms and syndromes in CM data analysis is also important.

Conventional methods usually use only one numerical value to describe the relationship of two symptoms. In this study, we use a pair of characteristic values to describe a relative link between the symptoms as a relative associated density (RAD). By analysing the characteristic value pairs, we searched significant one-way links between symptoms and confirmed the links according to CM theory [12,13]. The RAD method was also used to find one-way links among multiple syndromes in the clinical data.

Among a large number of symptoms in CM diagnosis data sets for a certain disease, some symptoms may be redundant. Therefore, selecting major or relevant symptoms is crucial to the performance of machine learning. Wang *et al*. [14] used support vector machine (SVM) to generalize symptom weights in CHD predictions. Liu *et al*. [15] used symptom frequency analysis to enhance modelling results in learning. Zhou *et al*. [16] developed a clinical reference information model (RIM) and a physical data model to manage various entities and relationships in CM clinical data. Principal component analysis (PCA) [17], partial least squares (PLS) [18], maximum relevance and minimum redundancy (MRMR) [19] have been used to perform symptom selection to improve prediction accuracy.

The results from conventional primary symptom selection or reduction methods are difficult to be interpreted in CM. For instance, PCA reduces symptom dimensionality at the expense of loss of medical meaning [20]. Although MRMR can predict fairly using only a few major symptoms [21], the results are often inconsistent with basic CM theory [12,13]. This study aims to use RAD to perform symptom selection, and evaluate whether the results can be better explained by CM theory [12,13].

## Methods

### Data set of CHD in CM

A total of 555 clinical cases were collected from the cardiology departments of Longhua Hospital, Shuguang Hospital, Shanghai Renji Hospital, and Shanghai Hospital of CM form March 2007 to May 2008 to compile the CHD data set used in this study. It could be obtained from the address http://levis.tongji.edu.cn/gzli/publication.htm[15].

Out of the 555 cases, 265 patients (47.7%) were male, age (mean ± standard deviation): 65.15 ± 13.17 and 290 patients (52.3%) are female, age: 65.24 ± 13.82. The symptoms collected from inquiry diagnosis include 125 symptoms in eight dimensions (cold or warm, sweating, head, body, chest and abdomen, urine and stool, appetite, sleeping, mood, and gynecology). The differentiation diagnosis includes 15 syndromes, as described in Liu *et al*. [15].

For unification of the results, specific types and feeling information of some symptoms were combined and some symptoms unique to females were deleted. The variables analyzed in this study include 63 symptoms and 10 syndromes. The 63 included symptoms were listed in Table 1. The 10 included syndromes were (I) heart-*qi *deficiency syndrome; (II) heart-*yang *deficiency syndrome; (III) heart-*yin *deficiency syndrome; (IV) heart-blood deficiency syndrome; (V) turbid phlegm syndrome; (VI) blood stasis syndrome; (VII) *qi *stagnation syndrome; (VIII) heart-fire hyperactivity syndrome; (IX) heart-kidney *yang *deficiency syndrome; (X) cardiopulmonary-*qi *deficiency syndrome.

**Table 1:** The 63 symptoms in the data set

**No**.	Symptom
1	Chills

2	Cold limbs

3	Dampness-heat

4	Feverish palms and soles

5	Spontaneous sweating

6	Night sweat

7	Palpitation

8	Chest distress

9	Chest pain

10	Short breath/dyspnea/suffocation

11	Edema

12	Hypodynamia

13	Dysphoria

14	Paroxysmal night dyspnea

16	Amnesia

16	Dizziness

17	Tinnitus

18	Mouth and tongue sore

19	Cough

20	Cough with sputum

21	Hiccup

22	Acid regurgitation

23	Gastric stuffiness

24	Gastralgia

25	Epigastric upset

26	Nausea and vomiting

27	Heavy breathing

28	Lateral thorax distending pain

29	Abdomen distending pain

30	Soreness and weakness of waist and knees

31	Numbness of hands and feet

32	Body soreness

33	Thirsty and dry pharynx

34	Absence of thirst and no desire for water drink

35	Intake of fluid failing resolve thirst

36	Like cold drink

37	Like hot drink

38	Poor appetite and less amount of food

39	Always hungry

40	Hunger without desire to eat

41	Bitter taste

42	Mucosity in mouth

43	Tastelessness in mouth

44	Loose stool

45	Water like stool

46	Diarrhea with undigested food

47	Diarrhea in the morning

48	Stool sometimes sloppy and sometimes bound

49	Constipation

50	Dry stool like sheep feces

51	Non-smooth defecation or tenesmus

52	Clear urine in large amounts

53	Dark urine

54	Frequent micturition

55	Deficient urine

56	Stranguria

57	Urinating burning heat

58	Dribble of urine

59	The frequent and increased urination at night

60	Aggravating gloom

61	Sleepiness

62	Impetuosity and susceptibility to rage

63	Easily frightened and scared

### The RAD method

#### Probability and statistics

In the medical diagnosis of CHD, frequency of symptom occurrence may be different. For instance, the chest tightness symptom and the dizziness symptom are frequent symptoms, while the sleepiness symptom and the diarrhea with undigested food symptom are rare symptoms. In the data analysis, the first step is to distinguish between the frequent and the rare symptoms.

In probability of symptoms, *Pf_i _*stands for the appearance probability of the *i*th symptom across all cases, which is defined as

(1) $$Pf_{i}=\frac{\sum_{m=1}^{N}F_{im}}{N}$$

where *F_im _*= 1 if the *i*th symptom appears in the *m*th case, or else *F_im _*= 0. *N *denotes the number of the cases.

Similarly, *Pl_i _*stands for the appearance probability of the *i*th syndrome across all cases, which is defined as

(2) $$Pl_{i}=\frac{\sum_{m=1}^{N}L_{im}}{N}$$

where the *i*th syndrome appears in the *m*th sample, *L_im _*= 1, or else *L_im _*= 0.

#### Building the symptom-symptom interaction network

Equations (1) and (2) calculate the appearance probability of all symptoms and syndromes. But these values cannot reveal their potential connections. Symptom-symptom interaction (SSI) network in the same manner as used for human social networks was used to find the connections [21,22].

When two different symptoms occur simultaneously in the same case, sign *G_im _*= 1 indicating that symptom *F_i _*and symptom *F_j _*appear at the same time in the *mth *case, or else *G_ijm _*= 0. *F_i_F_j _*stands for the number of simultaneous occurrences of *F_i _*and *F_j_*. Then for N cases,

(3) $$F_{i}F_{j}=\sum_{m=1}^{N}G_{ijm}$$

which contains two types of information: the frequency of features and the relevancy of two features.

### Relative associated density

Equation (3) is largely concerned with the frequency of symptoms. In other words, frequent relationships between symptoms are obvious, while less frequent relationships are hard to be detected. The difference is even more than 300 folds. Therefore, this study used RAD, which uses conditional probability to measure the relationships of symptoms and syndromes.

The term *C*(F_i_, F_j_) represents the RAD values of symptom *F_i _*associated with *F_j _*and use *C*(F_j_, F_i_) represents the RAD values of symptom *F_i _*associated with *F_j_*. According,

(4a) $$C\left(F_{i},\left(F_{j}\right)\right)=\frac{F_{i}F_{j}}{\sum_{m=1}^{N}F_{im}}$$

(4b) $$C\left(F_{j},\left(F_{i}\right)\right)=\frac{F_{i}F_{j}}{\sum_{m=1}^{N}F_{jm}}$$

#### Symptom selection with RAD

In the *mth *case, if symptom *F_i _*appears with syndrome *L_j_, H_ijm _*= 1; otherwise, *H_ijm _*= 0. Then for all N cases,

(5) $$F_{i}L_{j}=\sum_{m=1}^{N}H_{ijm}$$

RAD estimates the influence of the appearance probability on the interaction between a symptom and a syndrome. Equation (6) calculates the RAD value between symptoms and syndromes,

(6) $$\text{C}\left({\text{F}}_{\text{i}},\left(L_{j}\right)\right)=\frac{F_{i}L_{j}}{\sum_{m=1}^{N}L_{jm}}$$

This kind of association could be recognized as the contribution of one symptom to the syndrome.

Each syndrome was considered a single label; thus we selected corresponding symptoms regardless of their RAD values. For each single label prediction, the symptoms with low RAD values were removed one by one, and the predictions were calculated with SVM and KNN. The symptoms that lead to the highest prediction were recorded as the result of symptom selection.

MRMR symptom selection was used for a comparison [19]. The idea of MRMR is to search the optimal subset by maximizing relevance while minimizing redundancy based on mutual information. To maintain consistency with the RAD method, we used SVM [23] and KNN [24] for classification.

To evaluate the prediction results, we calculated the true positive rate (TPR), and true negative rate (TNR) criteria: TPR = TP/(TP + FN), TNR = TN/(FP + TN), where TP is the number of true positives, TN is that of true negatives, FP is that of false positives, and FN is that of false negatives. The G-means criterion was used to describe the equilibrium of the positive and negative classes of the prediction results, where G-means = (TPR * TNR)^1/2^.

## Results and discussion

RAD performed better than MRMR in feature selection for machine learning to discover CM relationships among the symptoms, syndromes, and even between the symptoms and syndromes in a CHD data set. RAD analysis found one-way connections among symptoms and the syndromes that are consistent with CM theory. RAD not only improves prediction accuracy but also enhanced interpretability.

### Common and rare symptoms

We used equation (1) to determine the symptom frequency in the data set. The first 20 frequent symptoms were identified as listed in Table 2. Table 3 lists the first 10 rare symptoms in the data set.

**Table 2:** The most frequent symptoms and their appearance probability

Order	Symptom	Appearance probability
1	Chest distress	78.6%

2	Short breath/dyspnea/suffocation	69.7%

3	Hypodynamia	65.4%

4	Palpitation	64.5%

5	Soreness and weakness of waist and knees	50.8%

6	Chest pain	48.6%

7	Thirsty and dry pharynx	48.6%

8	Dizziness	48.5%

9	Aggravating gloom	43.4%

10	Dysphoria	40.4%

11	Spontaneous sweating	39.1%

12	Numbness of hands and feet	37.1%

13	Night sweat	36.2%

14	Tinnitus	35.1%

15	Chills	35.0%

16	Cough	32.6%

17	Impetuosity and susceptibility to rage	32.3%

18	The frequent and increased urination at night	29.5%

19	Like cold drink	25.9%

20	Cough with sputum	25.4%

**Table 3:** The 10 rarest appeared symptoms and their frequency

Order	Symptom	Frequency
1	Urinating burning heat	0.2%

2	Sleepiness	0.7%

3	Diarrhea in the morning	0.9%

4	Hunger without desire to eat	1.1%

5	Non-smooth defecation or tenesmus	1.3%

6	Water like stool	1.4%

7	Diarrhea with undigested food	1.4%

8	Stool sometimes sloppy and sometimes bound	1.6%

9	Dribble of urine	2%

10	Always hungry	2.2%

**Figure 1:**
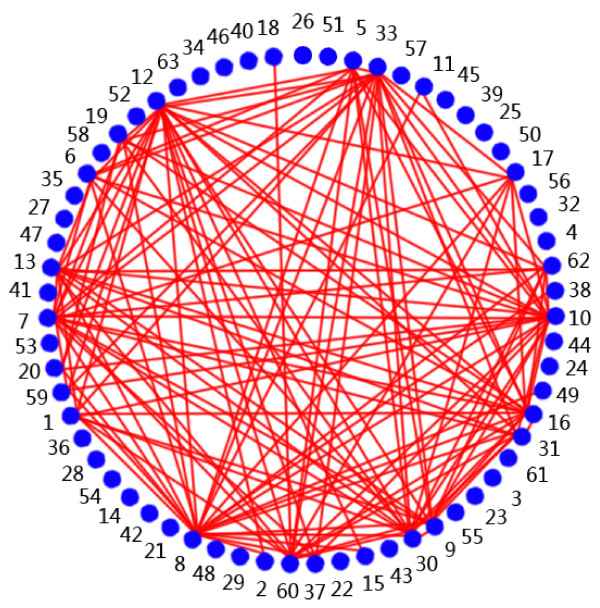
**The network of SSI**. The points denote the symptoms; solid lines connect the high SSI.

SSI was calculated by equation (3). Figure 1 shows a network constructed from the SSI results, *i.e*., the frequency and relationship among the symptoms. Table 4 lists the important symptoms shown in Figure 1.

**Table 4:** Symptoms with high SSI values shown in Figure 1

Symptom	Symptom
Tinnitus	Soreness and weakness of waist and knees

Spontaneous sweating	Thirsty and dry pharynx

Impetuosity and susceptibility to rage	Chills

Palpitation	Aggravating gloom

Numbness of hands and feet	Night sweat

Chest pain	Cough

Hypodynamia	Like cold drink

Dizziness	Cough with sputum

Short breath/dyspnea/suffocation	The frequent and increased urination at night

Dysphoria	Chest distress

CHD was identified as a kind of deficiency syndromes or excess syndromes. As shown in Tables 2 and 4, CHD was associated with kidney deficiency, diet disloyalty, mental disturbance, cold pathogen invasion, and other factors. CHD occurred in the heart but was related to the liver, the kidney, and the spleen. CHD was also bound with heart-*qi *deficiency, heart-*yang *deficiency, heart-blood deficiency, and heart-*yin *deficiency. The imbalance of liver, kidney, and spleen was often accompanied by turbid phlegm syndrome, *qi *stagnation syndrome, blood stasis syndrome. From the first 20 most frequent symptoms, the symptoms of chest distress, hard breath/dyspnoea/suffocation, palpitation, and chest pain were found to be the locating syndrome of syndrome patterns of the heart, in consistency with modern clinical practice of CHD in CM. Other symptoms among the top 20 were also basic factors in CM heart system diseases diagnosis [12,13].

Table 3 lists the top 10 rare symptoms and their probabilities. The symptoms of the heart syndrome patterns were hunger without desire to eat and water-like stool symptom. This result was also consistent with CM theory [12,13].

### Analysis using the RAD method

RAD analysis of the SSI networks was used to determine the connections between symptoms, and identified major symptoms in CHD.

Equation (4) was used to determine the RAD values of SSI, as shown in Table 5.

**Table 5:** Some RAD values of SSI

	*F_i_*						
	**Chills**	**Cold limbs**	**Dampness** **-heat**	**Spontaneous sweating**	**Palpitation**	**Chest distress**	**Chest** **pain**

*F_j_*							

Chills	0.0%	71.5%	28.8%	36.9%	41.1%	37.6%	38.1%

Cold limbs	45.4%	0.0%	22.0%	21.7%	25.7%	22.9%	23.3%

Dampness-heat	8.8%	10.6%	0.0%	15.7%	11.7%	11.0%	10.4%

Spontaneous sweating	41.2%	38.2%	57.6%	0.0%	41.9%	42.4%	41.5%

Palpitation	75.8%	74.8%	71.2%	69.1%	0.0%	68.6%	61.5%

Chest distress	84.5%	81.3%	81.4%	85.3%	83.5%	0.0%	80.7%

Chest pain	53.1%	51.2%	47.5%	51.6%	46.4%	50.0%	0.0%

*P_ij _*and *P_ji _*always appeared as a pair. Some symptoms were obviously one-way connections. For example, only 11.4% of occurrences of the hard breath symptom were accompanied by the hot flash symptom, while 74.6% of occurrences of the hot flash symptom appeared with the hard breath symptom. This was typical one-way connection between two symptoms.

Table 6 lists more connections between two symptoms. CM theory holds that chills occur with *yang *asthenia [12,13]. *Yin *asthenia occurs with hot flashes and night sweats [12,13]. The probabilities of chills appearing with hot flashes and night sweats is low, and their occurring probabilities are 0.087 and 0.061, separately.

**Table 6:** One-way connections between symptoms

Symptom	RAD (L to R)	Symptoms	RAD (R to L)
28 Lateral thorax distending pain	0.529	1 Chills	0.046

56 Stranguria	0.571	1 Chills	0.041

47 Diarrhea in the morning	0.600	3 Dampness-heat	0.050

28 Lateral thorax distending pain	0.529	5 Spontaneous sweating	0.041

42 Mucosity in mouth	0.764	5 Spontaneous sweating	0.059

43 Tastelessness in mouth	0.550	5 Spontaneous sweating	0.050

52 Clear urine in large amounts	0.625	5 Spontaneous sweating	0.046

53 Dark urine	0.571	5 Spontaneous sweating	0.055

42 Mucosity in mouth	0.529	6 Night sweat	0.044

14 Paroxysmal night dyspnea	0.933	7 Palpitation	0.078

25 Epigastric upset	0.826	7 Palpitation	0.053

35 Intake of fluid failing resolve thirst	0.700	7 Palpitation	0.058

Table 6 also lists the RAD values of one-way connections between symptoms. For instance, the probability of chills accompanied by body coldness was 71.5%, while the probability of body coldness accompanied by chills was only 45.4%. These unequal results indicate that a patient suffering from chills would be more likely to have the body coldness symptom. By contrast, a patient suffering from body coldness would be less likely to have the chills symptom. Furthermore, the locating symptom of chest distress occurred with qualitative and locating symptoms, such as paroxysmal night dyspnoea or orthopnoea, tastelessness and tediousness, nausea and vomiting, epigastric upset, deficient urine, dark urine, feverish palms and soles, intake of fluid failing to resolve thirst, stool resembling sheep's droppings. When paroxysmal night dyspnoea or orthopnoea happened, chest distress symptoms rarely appeared at the same time. Therefore, the one-way connections between the symptoms calculated by RAD explained the clinical results in CM. For example, *yang *asthenia was the representation of chills, and when chills present, distending pain in the hypochondrium and urine astringent pain appeared at the same time. However, the latter two symptoms did not represent chills; thus, they would not be accompanied by the symptom of chills. For another example, spontaneous sweating was an expression of the *qi *asthenia symptom and possibly appeared with distending pain in the hypochondrium, a sticky slimy sensation in the mouth, dark urine, but not vice versa. From these two examples, we can see that the contribution of chills to *yang *asthenia was greater than that of spontaneous sweating to *qi *asthenia. In the meantime, we may infer that distending pain in the hypochondrium, a sticky slimy sensation in the mouth, and dark urine are not typical features of *qi *asthenia and *yang *asthenia. This association analysis of symptoms can show which symptoms are major features and identify possible relationships between symptoms and syndromes. This kind of analysis would provide an objective basis for standardization of dialectic diagnosis.

### Relationships among the syndromes

Table 7 shows the frequencies of all 10 syndromes calculated using equation (2). Table 8 lists the RAD values of the syndrome.

**Table 7:** Frequency values of 10 syndromes

Order	Syndrome	Frequency
1	Blood stasis syndrome (VI)	76.0%

2	Heart-*qi *deficiency syndrome (I)	60.9%

3	Turbid phlegm syndrome (V)	48.3%

4	Heart-*yin *deficiency syndrome (III)	38.6%

5	Heart-*yang *deficiency syndrome (II)	31.4%

6	*Qi *stagnation syndrome (VII)	20.7%

7	Heart-kidney *yang *deficiency syndrome (IX)	11.7%

8	Heart-fire hyperactivity syndrome (VIII)	5.4%

9	Heart-blood deficiency syndrome (IV)	2.9%

10	Cardiopulmonary-*qi *deficiency syndrome (X)	2.5%

**Table 8:** RAD values of syndromes

					*L_i_*					
	**1**	**2**	**3**	**4**	**5**	**6**	**7**	**8**	**9**	**10**

***L_j_***										

1	0.00	0.01	0.81	0.69	0.62	0.64	0.59	0.60	0.03	0.71

2	0.01	0.00	0.08	0.06	0.33	0.30	0.27	0.10	0.97	0.00

3	0.51	0.10	0.00	0.13	0.44	0.38	0.31	0.60	0.09	0.43

4	0.03	0.01	0.01	0.00	0.03	0.02	0.02	0.03	0.00	0.00

5	0.49	0.50	0.55	0.44	0.00	0.55	0.50	0.43	0.46	0.79

6	0.80	0.73	0.75	0.63	0.87	0.00	0.84	0.53	0.63	0.86

7	0.20	0.18	0.17	0.13	0.22	0.23	0.00	0.33	0.11	0.14

8	0.05	0.02	0.08	0.06	0.05	0.04	0.09	0.00	0.02	0.07

9	0.01	0.36	0.03	0.00	0.11	0.10	0.06	0.03	0.00	0.00

10	0.03	0.00	0.03	0.00	0.04	0.03	0.02	0.03	0.00	0.00

### High correlation of the syndromes

Relevant analysis of the relationships between syndromes found high correlations in heart-*qi *insufficiency, such as heart-*yin *deficiency, heart-blood deficiency, turbid phlegm, blood stasis, *qi *stagnation, heart-fire hyperactivity, and cardiopulmonary *qi *deficiency. For example, blood stasis was highly correlated with heart-*qi *insufficiency, heart-*yang *insufficiency, heart-*yin *deficiency, heart-blood deficiency, turbid phlegm, *qi *stagnation, heart-kidney *yang *deficiency, and cardiopulmonary *qi *deficiency. The one-way RAD values of these syndromes were 0.80, 0.73, 0.75, 0.63, 0.87, 0.84, 0.63, and 0.86, respectively.

The finding of high correlation of heart-*qi *insufficiency with heart-blood deficiency and heart-*yin *deficiency is consistent with CM theory that a long period of heart-*qi *insufficiency would result in *yin *blood, causing fluid and blood deficiency and then *qi yin *deficiency [25]. In consistency with this theory, *qi yin *deficiency syndrome was common. The correlations of heart-*qi *insufficiency with turbid phlegm, blood stasis, *qi *stagnation, heart-fire hyperactivity, and cardiopulmonary *qi *deficiency were high, and consistent with the feature of deficiency syndrome or excess syndrome of CHD [12,13]. According to CM theory [12,13], turbid phlegm, *qi *stagnation, and blood stasis are symptoms, while *qi *deficiency is the radical that causes heart vessel stagnation and then CHD. The high RAD values of turbid phlegm and cardiopulmonary *qi *deficiency would explain that cardiopulmonary *qi *deficiency causes retention of water and dampness, and then sputum and more turbid phlegm [12,13].

The high degree of correlation of blood stasis with heart-*qi *insufficiency, heart-*yang *insufficiency, heart-*yin *deficiency, heart-blood deficiency, turbid phlegm, *qi *stagnation, heart-kidney *yang *deficiency, and cardiopulmonary *qi *deficiency indicates that blood stasis appeared in these syndromes. According to CM theory [12,13], heart controlling the blood vessel, *yang *asthenia, and *qi *asthenia may cause degradation of driving blood ability, and then blood stasis. Heart-fire hyperactivity and heat scorching blood viscous may cause blood stasis [12,13]. *Qi *stagnation and poor blood flow may also cause blood stasis [12,13]. Blood stasis may be the basic pathogenesis of CHD [26].

#### One-way connection of the syndromes

Table 8 shows some syndrome pairs with obvious one-way connections. For example, the RAD value of heart-*qi *insufficiency to insufficiency of the heart blood was 0.69, but the reversed RAD value was only 0.03. The RAD value of heart-*qi *insufficiency to heart-fire hyperactivity was 0.60, while the reversed RAD was 0.05. Table 9 summarizes the one-way connections of the syndrome pairs.

**Table 9:** One-way connections of the syndrome pairs

Syndrome	RAD (L to R)	Syndrome	RAD (R to L)
Heart-blood deficiency syndrome	0.687	Heart-*qi *deficiency syndrome	0.032
Heart-fire hyperactivity syndrome	0.600	Heart-*qi *deficiency syndrome	0.053
Cardiopulmonary-*qi *deficiency syndrome	0.714	Heart-*qi *deficiency syndrome	0.029
Heart-fire hyperactivity syndrome	0.600	Heart-*yin *deficiency syndrome	0.084
Cardiopulmonary-*qi *deficiency syndrome	0.785	Turbid phlegm syndrome	0.041
Heart-blood deficiency syndrome	0.625	Blood stasis syndrome	0.023
*Qi *stagnation syndrome	0.834	Blood stasis syndrome	0.227
Heart-fire hyperactivity syndrome	0.533	Blood stasis syndrome	0.037
Heart-kidney *yang *deficiency syndrome	0.630	Blood stasis syndrome	0.097
Cardiopulmonary-*qi *deficiency syndrome	0.857	Blood stasis syndrome	0.028

Taking heart-*qi *insufficiency and insufficiency of the heart blood as an example, CM theory [12,13] emphasizes the interdependence between *qi *and blood, and long-term *qi *insufficiencies will cause blood deficiency. However, insufficiency of the heart blood is not always accompanied by heart-*qi *insufficiency [12,13]. In elder patients, viscera function is weak, a pure sthenic syndrome is rare, and an asthenia with sthenia syndrome is more common. The RAD value of heart-*qi *insufficiency to heart-*yin *deficiency was 0.81, indicating that most CHD patients were *qi *asthenia together with *yin *asthenia. According to CM theory [12,13], heart-fire hyperactivity is not directly related to heart-*qi *insufficiency or insufficiency of heart-*yin*. High one-way connections were found for blood stasis to cardiopulmonary *qi *deficiency, insufficiency of the heart blood, heart-fire hyperactivity, *qi *stagnation, and heart-kidney *yang *deficiency. However, the RAD values of reversed connections were low, indicating that blood stasis was not the only reason for CHD.

#### Two-ways connections of the syndrome

In addition to the observations of one-way connections, two-way connections were also found. For example, the mutual RAD values of blood stasis and *qi *asthenia were 0.80 and 0.64, respectively, indicating that these two syndromes were highly correlated. CM theory [12,13] holds that *qi *asthenia and then poor blood flow would lead to blood stasis, in reverse. Long-term blood stasis may also cause *qi *asthenia. These two syndromes causally influence with each other.

### Relationships between symptoms and syndromes

According to CM theory [12,13], a symptom is an expression of internal syndrome, and a syndrome is essential to symptom appearance. The RAD results (Table 10) calculated by equation (6) showed the one-way connections of symptoms to syndromes, whose connections could be viewed as the contributions of symptoms to syndromes.

**Table 10:** Some RAD values between symptoms and syndromes

				Symptom		
**Syndrome**	**Chills**	**Cold** **limbs**	**Night** **sweat**	**Palpitation**	**Chest** **distress**	**Chest** **pain**

Heart-*qi *deficiency	0.260	0.127	0.367	0.627	0.790	0.441

Heart-*yang *deficiency	0.592	0.437	0.310	0.684	0.782	0.546

Heart-*yin *deficiency	0.294	0.182	0.509	0.696	0.827	0.453

Heart-blood deficiency	0.250	0.250	0.250	0.625	0.750	0.250

Turbid phlegm	0.354	0.239	0.373	0.701	0.802	0.522

Blood stasis	0.348	0.216	0.344	0.652	0.787	0.512

*Qi *stagnation	0.374	0.235	0.400	0.670	0.739	0.522

**Figure 2:**
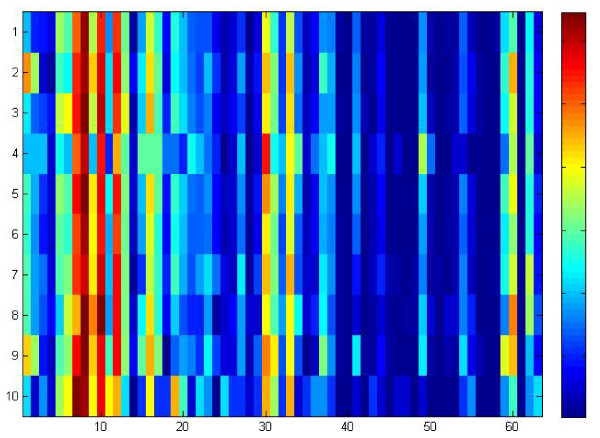
**The RAD values of symptoms to syndromes**.

Figure 2 illustrates the data in Table 10, where the x-axis represents the 63 symptoms and the y-axis represents the 10 syndromes. Red rectangles represent high RAD values, and the blue ones represent low RAD values. From Figure 2, the correlations between symptoms and syndromes were determined. As shown in Figure 2, the symptoms of palpitation, chest distress, short breath, weakness, soreness, and weakness of waist and knees were related to most of the syndromes. At the same time, chills and some other symptoms showed strong connections to some syndromes, such as heart-kidney *yang *deficiency and *yang *asthenia. Table 11 lists the symptoms and syndromes with high and low RAD values. In Table 11, chills showed a low relation to most of the syndromes except for heart-*yang *insufficiency and heart-kidney *yang *deficiency, indicating that chills were closely related to the latter syndromes. CM theory [12,13] holds that weakness of *yang *and *qi *and lack of warmth may cause chills. The high RAD values of night sweats to insufficiency of heart-*yin *did confirm the CM theory that *yang *cannot be restricted by *yin *asthenia, and then deficiency fire will be an internal disturbance and cause night sweats [12,13]. Constipation and insufficiency of heart blood showed a strong connection. *Inner Canon of Yellow Emperor *points out that "people over 40 years old may lose half of the *yin qi*", and CM theory [12,13] holds that insufficiency of the heart blood causes body fluid deficiency, which in turn causes insufficient lubrication of the colon, leading to constipation. The strong connections between nocturnal frequent micturition and heart-kidney *yang *deficiency can be explained by the lack of *yang *in the heart and kidney which resulted in a decrease of the controlling and *qi *transformation functions, bladder retention failure, and then nocturnal frequent micturition.

**Table 11:** Symptoms with relative high and low RAD values to syndromes

Symptom	Syndrome
Strong relation	

Chills	Heart-*yang *deficiency syndrome, Heart-kidney *yang *deficiency syndrome

Night sweat	Heart-*yin *deficiency syndrome, Cardiopulmonary-*qi *deficiency syndrome

Cough	Cardiopulmonary-*qi *deficiency syndrome

Soreness and weakness of waist and knees	Heart-blood deficiency syndrome

Constipation	Heart-blood deficiency syndrome

The frequent and increased urination at night	Heart-kidney *yang *deficiency syndrome

Edema	Cardiopulmonary-*qi *deficiency syndrome

Chest pain	Heart-blood deficiency syndrome

Weak relation	

The frequent and increased urination at night	Heart-blood deficiency syndrome, Cardiopulmonary-*qi *deficiency syndrome

Edema	Heart-blood deficiency syndrome

The weak connections (Table 11) of chest pain and insufficiency of the heart blood, nocturnal frequent micturition and insufficiency of the heart blood, and edema and insufficiency of the heart blood were also significant and consistent with CM theory [12,13].

### Symptom selection with RAD

In this study, RAD was used for symptom selection, and then SVM [23] and K-nearest neighbours (KNN) [24] were used for the prediction.

Table 11 shows individual contributions of symptoms to the syndromes.

The predictions were not sound as the syndromes 4, 8, 9, and 10 in this data set showed serious imbalance; therefore, we omitted these results. For syndromes 1, 2, 3, 5, 6, and 7, (Table 12), the results were much better. Table 12 indicates that the prediction results with MRMR favoured either the positive class or the negative class. In the G-means results of the syndromes, these maximum values were obtained by the RAD method, indicating that RAD achieved a good balance between the positive class and the negative class. Although for some syndromes, the prediction results of RAD and MRMR were close when the TPR, TNR, and G-means values were all considered. In general, the results obtained by RAD were more reasonable.

**Table 12:** Statistical Results of TPR, TNR and G-means by using SVM and KNN with RAD and MRMR or without symptom selection

Syndrome		1	2	3	5	6	7	Average
No Symptom Selection- SVM	TPR	0.708	0.463	0.729	0.472	0.799	0.906	0.680
	
	TNR	0.411	0.770	0.535	0.602	0.516	0.667	0.583
	
	G-m	0.539	0.597	0.625	0.533	0.642	0.777	0.630

RAD-SVM	TPR	0.723	0.518	0.786	0.588	0.796	0.771	0.713
	
	TNR	0.429	0.781	0.547	0.536	0.592	0.865	0.609
	
	G-m	**0.557**	0.636	**0.656**	0.561	0.686	**0.817**	**0.652**

MRMR-SVM	TPR	0.955	0.337	0.131	0.412	0.955	0.020	0.468
	
	TNR	0.070	0.893	0.970	0.704	0.027	0.970	0.606
	
	G-m	0.259	0.549	0.356	0.539	0.161	0.141	0.334

No Symptom Selection- KNN	TPR	0.757	0.553	0.439	0.461	0.826	0.534	0.595
	
	TNR	0.380	0.795	0.732	0.657	0.673	0.784	0.670
	
	G-m	0.536	0.663	0.567	0.550	0.746	0.647	0.631

RAD-KNN	TPR	0.749	0.670	0.509	0.485	0.887	0.522	0.607
	
	TNR	0.391	0.712	0.729	0.663	0.704	0.851	0.706
	
	G-m	0.541	**0.691**	0.601	**0.567**	**0.790**	0.667	0.643

MRMR-KNN	TPR	1.000	0.401	0.170	0.354	0.942	0.161	0.505
	
	TNR	0.146	0.901	0.981	0.783	0.018	0.897	0.621
	
	G-m	0.382	0.601	0.409	0.526	0.130	0.379	0.405

## Conclusions

The RAD method is effective for CM clinical data analysis, particular for analysis of relationships between symptoms in diagnosis and generation of compact and comprehensible symptom feature subsets.

## Competing interests

The authors declare that they have no competing interests.

## Authors' contributions

GZL designed the study, supervised the data analysis, and organized discussion of the results. MYY designed the experiment and write the manuscript. SS dedicated in experiment results analysis and manuscript revision. YLW implemented the analysis method and performed the experiments. GPL participated into analysis implementation, data acquisition, and result discussion. All authors read and approved the final manuscript.
